# Unravelling γδ T-cell dysregulation in the gut and its implications for immune-mediated diseases

**DOI:** 10.1242/dmm.052439

**Published:** 2025-09-18

**Authors:** Dilys Santillo, Evangelos Bellos, Vanessa Sancho-Shimizu

**Affiliations:** ^1^ Section of Paediatric Infectious Disease, Department of Infectious Disease, Faculty of Medicine, Imperial College London SW7 2AZ, London, UK; ^2^ Centre for Paediatrics and Child Health, Faculty of Medicine, Imperial College London, London SW7 2AZ, UK; ^3^ Section of Virology, Department of Infectious Disease, Faculty of Medicine, Imperial College London, London SW7 2AZ, UK; ^4^ Human Genetics and Genomic Medicine, Faculty of Medicine, University of Southampton, Southampton SO16 6YD, UK

## Abstract

Multisystem inflammatory syndrome in children (MIS-C) is a rare condition associated with severe acute respiratory syndrome coronavirus 2 (SARS-CoV-2) infection and characterised by systemic inflammation and T-cell dysfunction. A subset of patients with MIS-C were found to harbour rare variants in the gene *BTNL8* that disrupt BTNL8-BTNL3 heterodimer formation, likely leading to inadequate γδ T-cell regulation and subsequent disrupted gut homeostasis. MIS-C shares clinical features with Kawasaki disease and similar mechanisms of pathogenesis with inflammatory bowel disease, despite these diseases being clinically distinct entities. We explore the common link between these diseases: the potentially critical role gut immunity plays in the initiation and persistence of disease through the tight regulation of γδ T cells via BTNL8 and BTNL3. Understanding the role of BTNL8 in the context of the overlap between these conditions may aid preventative measures and treatment of these conditions.

## Teaser

**Summary:** Gut immunity may play a critical role in the initiation and persistence of multisystem inflammatory syndrome in children, inflammatory bowel disease and Kawasaki disease. Understanding this common link could aid preventative measures and treatment of these conditions.

## Introduction

Multisystem inflammatory syndrome in children (MIS-C) is a condition affecting ∼3 in 100,000 children worldwide following severe acute respiratory syndrome coronavirus 2 (SARS-CoV-2) infection (Whittaker et al., 2020; Dufort et al., 2020; La Torre et al., 2023). The illness presents similarly to Kawasaki disease (KD), another heterogeneous inflammatory condition affecting children. Although the cause of KD remains unknown, multiple triggers – including bacterial, viral and fungal infections – have been proposed (Chang et al., 2014; Kang et al., 2022; Mofors et al., 2025; Nakamura et al., 2019). Despite MIS-C having more profound gastrointestinal involvement than KD, symptoms overlap, including fever, rash, cardiovascular dysfunction and systemic inflammation (Miller et al., 2020; Verdoni et al., 2020; Sancho-Shimizu et al., 2021; Hoste et al., 2021). Patients are typically seropositive for SARS-CoV-2, despite no active infection being detectable in the upper respiratory tract, and possess elevated inflammatory markers [C-reactive protein, TNFα (also known as TNF), interleukin (IL)-6] with distinct interferon gamma (IFNγ) and nuclear factor kappa B (NFκB) immune signatures (Sacco et al., 2022). Activation of T cells, monocytes and neutrophils has been observed in the acute phase of disease, with a specific polyclonal expansion of T-cell receptor beta variable 11-2 (TRBV11-2) CD4^+^ and CD8^+^ T cells (Moreews et al., 2021). This specific T-cell expansion supports the hypothesis that superantigen-mediated T-cell activation is a driving mechanism of MIS-C pathogenesis (Porritt et al., 2021; Moreews et al., 2021). The cause of MIS-C remains debated, with several mechanisms proposed, including T-cell exhaustion, SARS-CoV-2 antigen persistence, superantigen-mediated T-cell activation and, most recently, Epstein–Barr virus (EBV) reactivation consequent to elevated TGFβ (Consiglio et al., 2020; Beckmann et al., 2021; Porritt et al., 2021; Yonker et al., 2021; Hsieh et al., 2022; Goetzke et al., 2025). Meanwhile, genetic investigations into MIS-C susceptibility have revealed gene defects in variants – specifically in *OAS1*, *OAS2* and *RNASEL* – and in butyrophilin-like 8 (*BTNL8*), leading to aberrant inflammatory responses (Lee et al., 2023; Bellos et al., 2024). Autosomal recessive variants in the OAS-RNaseL pathway have been shown to lead to exacerbated cytokine production in mononuclear phagocytes, whereas variants in *BTNL8* are thought to dysregulate the BTNL8-γδ T-cell axis.

A shared genetic risk locus has been observed in inflammatory bowel disease (IBD), a condition resulting in chronic intestinal inflammation, whereby a *BTNL8-BTNL3* copy number variant (CNV) has been identified as a risk modifier for disease (Dart et al., 2023). This results in a 56 kb deletion, resulting in a fusion protein lacking the extracellular domain of BTNL3, thus driving the T-cell dysfunction and the profound phenotype observed (Dart et al., 2023; Aigner et al., 2013). Despite KD, IBD and MIS-C being clearly distinct clinical entities, they share similar features of disease (MIS-C and KD) and mechanisms of pathogenesis (MIS-C and IBD) via gut dysregulation, which may predispose to a hyper-inflammatory state (Table 1). Understanding the overlap between these inflammatory diseases could aid preventative measures and treatment of these conditions. In this Perspective, we aim to draw connections between these inflammatory diseases to propose future research that could provide insights into multiple conditions.

Understanding the overlap between these inflammatory diseases could aid preventative measures and treatment of these conditions

**Table 1.** Comparison of characteristics of Kawasaki disease, multisystem inflammatory syndrome in children and inflammatory bowel disease

	KD	MIS-C	IBD
Incidence	∼9 in 100,000 (Odingo et al., 2023)	∼3 in 100,000 (La Torre et al., 2023)	0.2-46.1 in 100,000 (Caron et al., 2024)
Presentation	Systemic disease with occasional gut involvement	Systemic disease with gut involvement	Systemic disease that primarily affects the gut
Type of condition	Acute inflammatory response with isolated incidence	Acute post-viral inflammatory response with isolated incidence	Autoinflammatory disorder; chronic inflammation with relapse
Trigger	Unknown – multiple pathogens proposed (Chang et al., 2014, Kang et al., 2022, Mofors et al., 2025, Nakamura et al., 2019, Burns, 2024)	SARS-CoV-2 (Whittaker et al., 2020)	Diet, microbiome, stress, infection, medication (Ananthakrishnan, 2013, Mann and Saeed, 2012, de Souza et al., 2017)
Age of presentation	Children (median age <5 years)	Children (median age 9 years)	All ages
Treatment	Immunosuppressive therapies (steroids and IVIG)	Immunosuppressive therapies (steroids and IVIG)	Anti-inflammatory, immunosuppressive therapies and surgery
Outcome	Life threatening with potential life-long cardiac complications	Life threatening but likely full recovery if treated	Rarely life threatening but can severely impact quality of life

IBD, inflammatory bowel disease; IVIG, intravenous immunoglobulin; KD, Kawasaki disease; MIS-C, multisystem inflammatory syndrome in children; SARS-CoV-2, severe acute respiratory syndrome coronavirus 2.

## T-cell dysregulation in the gut and the role of BTNL8

*BTNL8* is a gene that has been implicated in both MIS-C and IBD, albeit in different ways. BTNL8 is a transmembrane protein expressed on the surface of healthy intestinal epithelial cells in complex with BTNL3 (Chapoval et al., 2013; Di Marco Barros et al., 2016; Vantourout et al., 2018; Maynard and Weaver, 2009). This complex specifically engages a subset of γδ T cells, notably Vγ4^+^ intraepithelial lymphocytes (IELs) (Willcox et al., 2019; Melandri et al., 2018). γδ T cells are a unique class of T lymphocytes characterised by their heterodimeric γ and δ chains (Li et al., 2023). They are generally activated in a major histocompatibility complex (MHC)-independent manner and directly recognise various antigens, including phospho and lipid antigens (CD1D), and stress molecules including toll-like receptor ligands (Vantourout and Hayday, 2013). These cells are predominantly tissue resident and are enriched in the intestines, the dermis and the lungs. Vγ4^+^ T cells make up a large proportion of the IELs and have been implicated in proinflammatory responses producing IL-17 and IFNγ (Vantourout and Hayday, 2013). Dysregulation of T-cell populations, including γδ T cells, has been reported in both coronavirus disease 2019 (COVID-19) and MIS-C, with studies noting reduced peripheral blood γδ T-cell numbers in patients with MIS-C (Carter et al., 2020). It is hypothesised that, in the healthy gut environment, the BTNL8/BTNL3 complex acts as a ‘normality signal’, maintaining immune tolerance and preventing inappropriate activation. Upon inflammation or infection, BTNL8 and BTNL3 expression is often reduced or absent. This loss of signal is likely detected by γδ T cells, which then become activated and may initiate immune surveillance or attack. Although this has not been shown in the gut, it has been observed to some extent with Skint1, another BTNL protein, involved in regulating γδ T cells in the skin of mice (McKenzie et al., 2022).

### BTNL8 and MIS-C

A recent gene burden analysis in patients with MIS-C revealed enrichment for rare damaging variants in *BTNL8* (odds ratio, 4.2; 95% confidence interval, 3.5-5.3; *P*<10^−6^) (Bellos et al., 2024). Of the 25 variants identified in MIS-C, eight were hypomorphic based on an *in vitro* assay testing surface expression, T-cell receptor (TCR) engagement and T-cell activation, accounting for 2.3% of patients (Bellos et al., 2024). The hypomorphic variants identified displayed a reduction in BTNL8 surface expression, thus reducing availability of BTNL3 for the Vγ4 ligand and providing a likely reason for the impaired engagement of the protein with Vγ4^+^ γδ T cells, disrupting TCR downregulation. Although these variants are hypomorphic *in vitro*, they may phenocopy what is seen with the CNV in IBD *in vivo*; however, this remains unknown. The variants identified were evenly distributed throughout the protein, but the majority of those with reduced function were located within the intracellular B30.2 domain responsible for forming the BTNL8-BTNL3 heterodimer (Bellos et al., 2024). Although these variants are not predicted to impact heterodimer formation with BTNL3, there may be other proteins that BTNL8 can interact with via its B30.2 domain that remain unknown. Furthermore, the individuals with MIS-C also showed altered intestinal permeability and elevated plasma zonulin, a marker for intestinal integrity (Bellos et al., 2024; Wang et al., 2000; Fasano et al., 2000). SARS-CoV-2 has shown to cause dysbiosis through excessive IL-6-mediated zonulin release, which is likely to explain the increased intestinal permeability observed (Yonker et al., 2021; Zari et al., 2024). γδ T cells play a crucial role in maintaining gut homeostasis, disruption of which can result in both localised inflammation and a widespread systemic response (Nielsen et al., 2017; Chen et al., 2002). It is likely that the viral trigger disrupts gut homeostasis, leading to prolonged inflammation in the individuals with *BTNL8* variants owing to poor restoration of gut immunity, similar to what is hypothesised in IBD patients (Dart et al., 2023).

### BTNL8 and IBD

Variants in *BTNL8* have also been implicated in IBD. IBD refers to a group of diseases involving chronic inflammation of the gut and primarily encompasses Crohn's disease (CD) and ulcerative colitis, affecting 3-20 in 100,000 and 0.5-31.5 in 100,000, respectively (da Silva et al., 2014; Feuerstein and Cheifetz, 2017). An imbalance in the microbiota, coupled with excessive activation of effector T cells, is thought to contribute to proinflammatory cytokine production, leading to chronic inflammation (Maynard and Weaver, 2009; Qiu et al., 2022). However, genetic susceptibility has been reported as a risk factor for disease (Maynard and Weaver, 2009; Gomez-Bris et al., 2023; Jarmakiewicz-Czaja et al., 2022; Dart et al., 2023). BTNL8 has been associated with CD severity, with a common CNV polymorphism implicated as a risk modifier for the disease (Dart et al., 2023). The CNV leads to the production of a hypomorphic BTNL8-BTNL3 fusion protein lacking the B30.2 domain of BTNL8 and IgV domain of BTNL3. Given the importance of the extracellular interaction with BTNL3 and the Vγ4 TCR, the resultant protein cannot interact with Vγ4^+^ T cells, rendering the protein non-functional (Dart et al., 2023). Individuals with the CNV genotype display a more profound IBD phenotype, with reduced Vy4^+^ cells from the CD103 compartment and phenotypically different Vy4^+^ T-cell populations, expressing higher levels of CD5 (Dart et al., 2023). Although BTNL8 is implicated in IBD, it is likely that the effect of the CNV on γδ T cells is also caused by the complete absence of BTNL3. This is dissimilar to patients with MIS-C, who do not have any defect in BTNL3. However, the CNV does not impact the baseline state of intestinal epithelial cells in patients with IBD, and this is likely the case for the variants identified in MIS-C, but it may impair the ability to resolve the triggered inflammation. Vγ4^+^ T-cell populations were not studied in the patients with MIS-C with the *BTNL8* variants, so it would be of interest to investigate their Vγ4^+^ T-cell populations to see whether these differ like they do in individuals homozygous for the BTNL8-BTNL3 CNV (Dart et al., 2023). Furthermore, certain TCR gamma variable 4 (TRGV4) polymorphisms expressed on Vγ4^+^ T cells have been shown to hinder BTNL3 binding, reducing TCR downregulation (Corcoran et al., 2023). It would be interesting to determine whether any of the patients with MIS-C carry these alleles as this may offer another explanation for the phenotype observed in the individuals without *BTNL8* variants. Additionally, assessing whether they would be more at risk for developing or presenting with IBD in the future compared to those without hypomorphic variants would be another particularly relevant question to follow up on.

## Expanding BTNL8 investigations

So far, only specific TCR function has been tested in a heterologous overexpression system (Bellos et al., 2024); therefore, future studies would benefit from exploring other potential functions of BTNL8. *BTNL8* is highly expressed in the gut with reports of expression also in the blood, specifically in neutrophils (https://www.gtexportal.org/home/gene/BTNL8). However, there has been no functional follow up, leaving the role of BTNL8 in the blood unknown. BTNL8 may have other functions, potentially independent of BTNL3 and beyond γδ T cells, that are currently unexplored, especially given that *BTNL3* mRNA and protein expression in the blood is negligible (https://www.proteinatlas.org/ENSG00000168903-BTNL3; https://www.gtexportal.org/home/gene/BTNL3). This is something that warrants further investigation as there may be cell type specificity to BTNL8 function, and it would be of interest to determine whether patient neutrophils display phenotypic or functional abnormalities. Additionally, BTNL8 has been described as a possible marker for macrophage-induced IL-10-expressing induced regulatory T cells. These are a subset of regulatory T cells expressing the anti-inflammatory cytokine IL-10 that are produced in response to macrophage signalling (Riquelme et al., 2018). This suggests another potential role for BTNL8 in the blood. Furthermore, genetic or proteomic exploration of BTNL8 interacting partners may reveal further molecular functions of BTNL8.

Current investigations in mouse models into butyrophilin-like proteins have revealed their role in shaping the local T-cell population and have highlighted the importance of heterodimerisation in their activation (Di Marco Barros et al., 2016). Mouse models have been very informative; however, biopsy-derived intestinal organoids with loss-of-function *BTNL8* variants co-cultured with Vγδ^+^ T cells would be an ideal system in which to recreate a full model of the intestine. Biopsy-derived intestinal organoids are regarded as more physiologically relevant, faster to generate and better suited for disease modelling than mouse models as they carry the desired patient genotype (Hautefort et al., 2022). Alternatively, induced pluripotent stem cell-derived organoids could be generated in a less invasive manner as peripheral blood mononuclear cells can be reprogrammed into intestinal cells without the need for a gut biopsy (McCracken et al., 2011; Vlahos et al., 2019). They also offer the added advantage of allowing reversal of cell polarity depending on the desired downstream investigations (Kromann et al., 2024; Kakni et al., 2022). However, these organoid models are likely to require more time and validation to fully mimic the native intestine. Although these organoid models would harbour the desired variants from the patient, a previous study of intestinal organoids from IBD patients exhibited a reversion towards a healthier phenotype after a few weeks in culture, suggesting that additional elements, such as lymphocytes, chemokines and dietary factors, are required to fully recapitulate the phenotype in organoid cultures (Arnauts et al., 2020). We propose that further insights into the pathogenic role of *BTNL8* variants in both MIS-C and IBD could be gained from further experiments in this area.

Despite KD, IBD and MIS-C being distinct clinical entities, the potential role of gut immunity could be the common link between these diseases

## Implications for KD

KD is an inflammatory condition, similar to MIS-C, affecting 9 in 100,000 children under the age of 5 years (Odingo et al., 2023). The aetiology of KD remains unclear; however, it is thought that an infectious trigger in individuals who are genetically predisposed initiates an aberrant immune response (Dietz et al., 2017; Burns, 2024). Although MIS-C and KD are both paediatric inflammatory disorders, they were originally characterised by distinct triggers, laboratory results, demographics and epidemiology (Sancho-Shimizu et al., 2021; Hoste et al., 2021). However, similar to MIS-C, we propose that gut immunity may also play a role in KD pathogenesis. Despite gastrointestinal symptoms being less frequent, gut microbial imbalance has been reported as a likely susceptibility factor for KD, with patients having a higher abundance of pro-inflammatory bacteria and lower proportion of bacteria known to suppress inflammation (Chen et al., 2020; Takeshita et al., 2002). Studies also show that gastrointestinal symptoms in KD are often liked to more severe outcomes and resistance to intravenous immunoglobulin therapy (Fabi et al., 2018). Moreover, increased intestinal permeability has been previously hypothesised as KD patients have elevated serum secretory IgA and mouse models exhibit a downregulation of tight junction proteins including claudin-1, occludins and zonula occludens-1 (Wang et al., 2023; Noval Rivas et al., 2019). Although there are no known published findings implicating BTNL8 in KD, we observed increased plasma zonulin in our KD cohort, albeit in a small sample size, supporting the hypothesis of increased intestinal permeability, which could indicate a potential shared mode of pathogenesis between MIS-C and KD (Bellos et al., 2024).

The incidence of MIS-C decreased drastically from 1 in 4000 infected in 2020 to 1 in 10,000 in 2022 with the emergence of new SARS-CoV-2 variants (Holm et al., 2021; Lopez et al., 2022). This is likely due to increased natural or vaccine-induced immunity but may also be due to differences in virus-host interactions with the different variants (Cohen et al., 2023; Holm et al., 2021; Dufort et al., 2020; Lee et al., 2023). Patients that present with MIS-C typically display characteristics that are very similar to KD, making it harder to distinguish between the two phenotypes (Verdoni et al., 2020; Whittaker et al., 2020). However, owing to the similar treatment approaches for MIS-C and KD, such diagnostic ambiguity is unlikely to significantly impact immediate patient management. In fact, this convergence may even facilitate research into KD, a condition for which pathogenesis remains less clearly defined. Conversely, the prolonged sequelae of MIS-C remain to be determined owing to the recent emergence of the disease, and reports have been limited (Fremed and Farooqi, 2022; Penner et al., 2021). Adding to the diagnostic complexity, the TRBV11-2T cell expansion, which was a distinguishing feature of MIS-C, has now been observed in pre-pandemic samples in individuals with MIS-C-like phenotypes (Benezech et al., 2023). This may add to the confusion of diagnosis between MIS-C and KD but also suggests that other pathogens, such as seasonal coronaviruses or EBV, can trigger similar hyperinflammatory responses that resemble MIS-C (Benezech et al., 2023; Goetzke et al., 2025). By elucidating the common mechanisms underlying these inflammatory diseases, we can gain a deeper understanding of their implications for prevention, diagnostics and therapeutics.

## Conclusion

Despite KD, IBD and MIS-C being distinct clinical entities, the potential role of gut immunity could be the common link between these diseases. MIS-C and IBD share mechanisms of pathogenesis via dysregulation of the BTNL8-γδ T-cell axis, potentially predisposing patients to an inflammatory state. Similarities in genetic susceptibility highlight the critical roles of this pathway, and gut immunity more broadly, in these diseases. Conversely, MIS-C and KD do not have a known mechanism of pathogenesis in common, but they display similar features of disease. We propose that gut immunity may also be important in the development of KD, although further work is needed to elucidate this link. By understanding the overlap between inflammatory diseases with shared genetic modulators, we can better ascertain the implications for other diseases with similar phenotypes.
